# Case Report: Neurogenic pulmonary edema coupled with myocardial damage following tenecteplase thrombolysis for acute ischemic stroke

**DOI:** 10.3389/fcvm.2026.1703463

**Published:** 2026-02-17

**Authors:** Xiaowei Li, Haixia Mo, Huixin Yang, Huijuan Shi, Jiannan Chen

**Affiliations:** 1 Department of Neurology, Xinle Hospital, Shijiazhuang, China; 2 Department of Neurology, Hebei Medical University Third Hospital, Shijiazhuang, China

**Keywords:** catecholamine storm, ischemic stroke, myocardial damage, neurogenic pulmonary edema, peripheral organ damage

## Abstract

Neurogenic pulmonary edema (NPE) is defined as an acute respiratory distress syndrome triggered by severe sympathetic discharge from acute compromise in the central nervous system. Due to the absence of specific diagnostic indicators, NPE is challenging to rapidly identify and accurately diagnose. We report a case of NPE coupled with myocardial damage following tenecteplase thrombolysis for acute ischemic stroke, explore the differential diagnosis of pulmonary edema after thrombolysis, and analyze multiple peripheral organ damage following ischemic stroke. A 61-year-old Chinese male presented with acute-onset aphasia and left-sided hemiparesis persisting for 3.25 h. Approximately one hour after tenecteplase thrombolysis, the patient developed acute respiratory distress. Arterial blood gas analysis demonstrated PaO₂ of 54 mmHg and PaCO₂ of 62 mmHg. Chest CT revealed patchy opacities with indistinct margins in the dorsal lung regions. Laboratory investigations revealed elevated levels of creatine kinase, creatine kinase-MB, B-type natriuretic peptide, and high-sensitivity troponin I. Echocardiogram demonstrated reduced left ventricular systolic function. The electrocardiogram exhibited ST-T segment abnormalities. Abnormal laboratory parameters indicative of hepatic and renal dysfunction were observed on admission day 2. Based on the clinical diagnosis of NPE, therapeutic interventions were initiated, including intravenous administration of furosemide, methylprednisolone sodium succinate, sodium nitroprusside, and piperacillin sodium/tazobactam sodium. The patient's clinical manifestations, laboratory parameters, and imaging findings improved significantly. Additionally, Takotsubo syndrome was considered as a potential comorbid condition. The hepatic and renal impairment were postulated to result from severe hypoxemia, and may also be modulated by neurohumoral mechanisms following massive cerebral infarction.

## Introduction

Neurogenic pulmonary edema (NPE), is defined as an acute respiratory distress syndrome (ARDS) triggered by severe sympathetic discharge secondary to acute central nervous system (CNS) compromise, and is characterized by rapid accumulation of pulmonary interstitial fluid (1). Common triggers include encephalitis, intracranial hemorrhage (including cerebral hemorrhage and subarachnoid hemorrhage), traumatic brain injury, epilepsy, cerebral infarction, and mass lesions involving the brain or spinal cord (2). Onset typically occurs within 4 h or is delayed (12–72 h) after CNS injury. Symptomatic resolution is observed within 72 h in approximately 50% of cases (3). Clinical manifestations comprise dyspnea, tachypnea, tachycardia, cyanosis, pink frothy sputum, and auscultatory findings of crackles or rales (1). Hypoxemia is evidenced by reduced PaO₂ and a PaO₂/FiO₂ ratio <200 mmHg, and chest radiography indicates bilateral infiltrates characterized by rapid resolution (3). Due to the lack of specific diagnostic markers, rapid recognition and accurate diagnosis remain challenging. Unfortunately, NPE can be fatal in critically ill patients if appropriate intervention is not administered. We report a case of NPE coupled with myocardial damage following tenecteplase thrombolysis for acute ischemic stroke, explore the differential diagnosis of pulmonary edema after thrombolysis, and analyze multiple peripheral organ damage following ischemic stroke.

## Case description

A 61-year-old Chinese male presented to the emergency department with acute-onset aphasia and left-sided hemiparesis persisting for 3.25 h. His medical history included hypertension, type 2 diabetes mellitus, and coronary artery disease. Chronic medications comprised aspirin, metformin, acarbose, amlodipine besylate, and nifedipine sustained-release tablets. Stroke code activation was initiated, with neurological examination revealing unresponsiveness to verbal commands, left central facial-lingual palsy, left hemiplegia (upper/lower limbs: Medical Research Council grade 1/3), and a positive left Babinski sign. The neurologist documented an initial National Institutes of Health Stroke Scale (NIHSS) score of 11. Vital signs showed blood pressure of 112/72 mmHg, pulse of 104 bpm, and respiratory rate of 20 bpm, and blood glucose level was 7.6 mmol/L. The patient underwent emergent brain CT imaging, which demonstrated multiple lacunar infarctions in the left cerebellar hemisphere, bilateral basal ganglia, and deep frontal and parietal lobes, with areas of encephalomalacia in the right cerebellar hemisphere and ischemic changes in the white matter. Given the patient's persistent neurological deficits and absence of contraindications, the medical team initiated thrombolytic therapy with tenecteplase at 0.25 mg/kg (total dose: 16 mg). Approximately one hour post-thrombolysis, during cranial DWI and MRA, the patient developed acute respiratory distress and was immediately transferred back to the ward. Physical examination revealed coma, with shallow and slow respirations accompanied by frothy sputum at the mouth, and absence of voluntary movement in all extremities. Vital signs were significantly deranged: heart rate 156 bpm, respiratory rate 26 bpm, and blood pressure 166/100 mmHg. Pulmonary auscultation revealed diffuse moist rales throughout both lung fields. Emergency endotracheal intubation was immediately initiated with mechanical ventilation. Subsequently, the patient expelled copious pink frothy sputum. Arterial blood gas (ABG) analysis demonstrated pH 7.04, PaO₂ 54 mmHg, O₂ saturation 72.2%, PaCO₂ 62 mmHg, HCO₃− 16.8 mmol/L, base excess −14.5 mmol/L, and lactate 7 mmol/L (PaO₂/FiO₂ ratio 257 mmHg). Cardiac enzyme assays revealed creatine kinase (CK) 1,286 U/L (reference: 50–310 U/L), CK-MB 33 U/L (reference: 0–25 U/L), and lactate dehydrogenase (LDH) 537 U/L (reference: 120–250 U/L). B-type natriuretic peptide (BNP) was elevated at 324.7 pg/mL (reference: 0–100 pg/mL), with high-sensitivity troponin I (hs-TnI) 221 pg/mL (reference: ≤100 pg/mL). Initial renal function, electrolytes, and coagulation profiles were within normal limits. (Note: Abnormal hepatic/renal indices emerged on admission day 2: creatinine 116 umol/L [57–111], alanine aminotransferase [ALT] 204.6 U/L [9–50], aspartate transaminase [AST] 317.7 U/L [15–40]; these parameters later trended downward with clinical improvement.) Portable chest radiography showed bilaterally increased and coarsened pulmonary markings with patchy opacities, while the cardiac silhouette appeared normal. Echocardiogram identified left atrial enlargement, discoordinated left ventricular wall motion, mild aortic regurgitation, and trivial mitral/tricuspid regurgitation, with reduced left ventricular systolic function [left ventricular ejection fraction (LVEF) 41%; Other echocardiogram parameters see Supplementary Table 1]. Electrocardiogram (ECG) displayed sinus rhythm at 97 bpm with ST-segment depression in leads I, II, aVF, and V4-V6 (QTc 443 ms). Given the diagnosis of acute pulmonary edema, therapeutic interventions included intravenous furosemide, methylprednisolone sodium succinate, sodium nitroprusside, and piperacillin sodium/tazobactam sodium.

**Figure 1:**
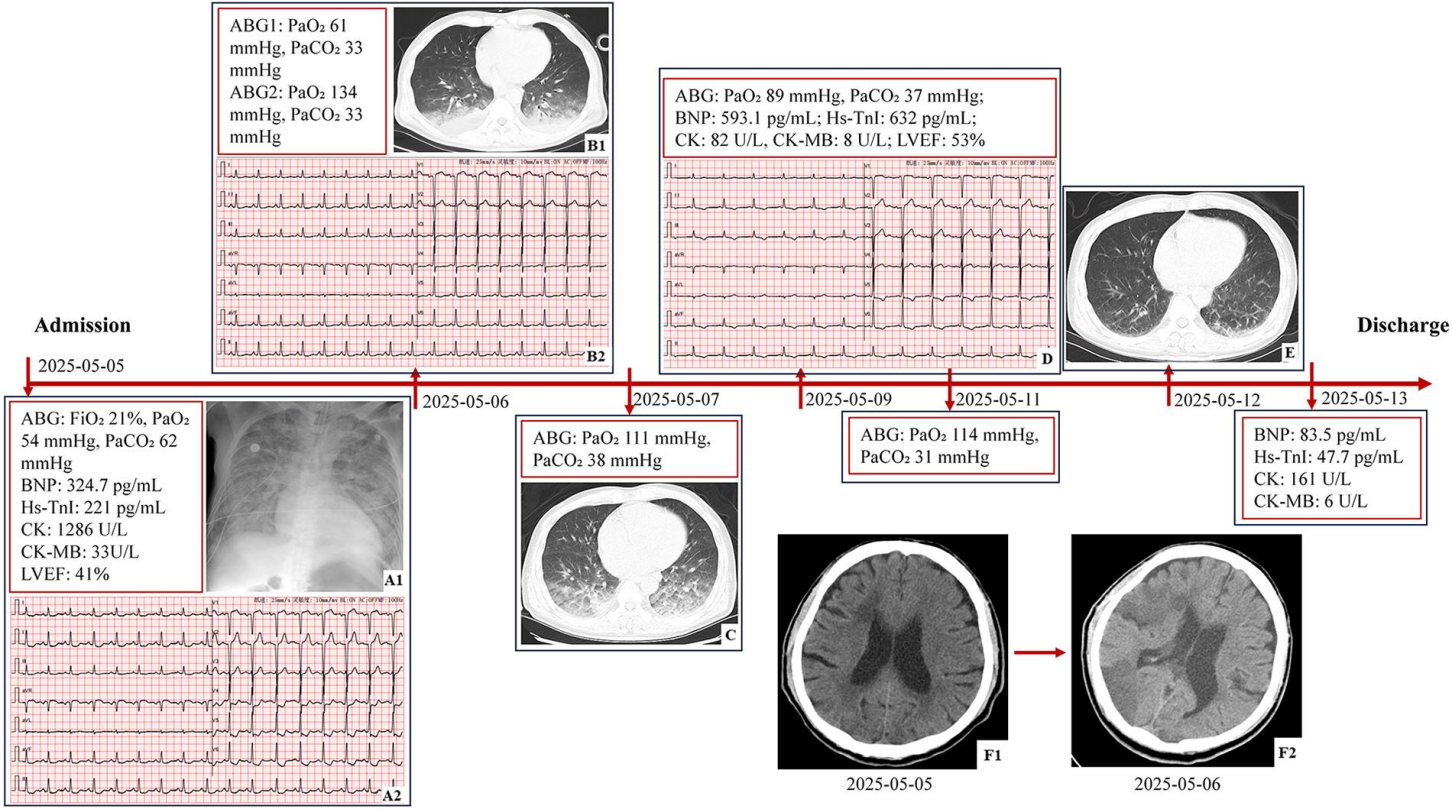
The timeline of findings from laboratory, imaging, and functional tests. **(A1)** Portable chest radiography: bilaterally increased and coarsened pulmonary markings with patchy opacities. **(A2)** ECG: sinus rhythm at 97 bpm, ST segment depression in leads I, II, aVF, and V4-V6, and QTc 443 ms. **(B1)** Chest CT: hyperlucency in bilateral upper lobes, patchy opacities with indistinct margins in dorsal lung regions, normal cardiac silhouette, small pericardial effusion, and bilateral pleural effusions. **(B2)** ECG: sinus rhythm at100 bpm, mild ST segment depression in leads V5 and V6, and QTc 390 ms. **(C)** Chest CT: partial resolution of edema. **(D)** ECG: sinus rhythm at 75 bpm, T wave inversion in leads II, III, aVF, V5, and V6, and QTc 438 ms. **(E)** Chest CT: near-complete resolution of edema. **(F1)** Brain CT: multiple lacunar infarctions in the left cerebellar hemisphere, bilateral basal ganglia, and deep frontal and parietal lobes, with areas of encephalomalacia in the right cerebellar hemisphere and ischemic changes in the white matter. **(F2)** Brain CT: extensive cerebral infarction involving the right frontal, temporal, occipital, parietal lobes, insular cortex, and basal ganglia region (ABG, arterial blood gas; hs-TnI, high-sensitivity troponin I; BNP, B-type natriuretic peptide; LVEF, left ventricular ejection fraction; CK, creatine kinase; CK-MB, creatine kinase-MB; ECG, electrocardiogram; QTc, corrected QT Interval).

On admission day 2, chest CT revealed bilateral upper lobe hyperlucency, patchy opacities with indistinct margins in dorsal lung regions, normal cardiac silhouette, small pericardial effusion, and bilateral pleural effusions. The patient's pulmonary edema symptoms improved significantly while sedated, with O₂ saturation consistently >98% and a marked reduction in pink frothy sputum production. Repeat cranial CT demonstrated extensive cerebral infarction involving the right frontal, temporal, occipital, and parietal lobes, insular cortex, and basal ganglia. Remaining findings were unchanged from the emergency CT. On admission day 3 (during analgesic administration), although the patient exhibited somnolence, he maintained normal verbal responsiveness and intact right limb voluntary movement. Concurrent chest CT demonstrated partial resolution of pulmonary edema, with ABG analysis remaining within normal limits. Clinical improvement continued, with repeat ABG analysis on day 5 showing pH 7.48, PaO₂ 89 mmHg, O₂ saturation 97.4%, PaCO₂ 37 mmHg, HCO₃− 27.6 mmol/L, base excess +4.0 mmol/L, and lactate 1.5 mmol/L. LVEF recovered to 53% (Supplementary Table 1). The tracheal tube was extubated on day 6. Transfer to the general neurology ward occurred on day 7 for specialized neurological and rehabilitation management. Follow-up chest CT on day 8 showed near-complete resolution of edema. Serum CK, CK-MB, BNP, and hs-TnI levels normalized by hospital day 9 (Figure 1).

## Discussion

The diagnosis of pulmonary edema is confirmed in this patient. The etiology, however, merits careful consideration, including potential neurogenic, cardiogenic, or medication-related (tenecteplase) causes. Advanced age, hypertension, cardiovascular disease, diabetes, hyperlipidemia, and smoking history are established risk factors not only for stroke but also for cardiac impairment. This presents a diagnostic challenge, as pulmonary edema in this context frequently results from combined neurogenic and cardiogenic mechanisms (1). Although distinguishing between cardiogenic and non-cardiogenic pulmonary edema proves clinically challenging, this differentiation remains essential for optimal therapeutic management.

The pathophysiology of NPE is initiated by increased intracranial pressure, which leads to sympathetic overstimulation or a catecholamine storm (4). This sympathetic surge causes systemic vasoconstriction, shunting blood from the systemic circulation to the pulmonary circulation. Pulmonary hypertension, combined with increased pulmonary capillary permeability, results in fluid accumulation in the alveolar and interstitial spaces and the subsequent development of pulmonary edema (5, 6). Additionally, the insular cortex (Ic) has been reported to play a critical role in regulating the autonomic nervous system (7, 8). The lateralization theory, positing that the right Ic primarily controls the sympathetic nervous system and the left Ic controls the parasympathetic nervous system, supports the view that sympathetic activation and NPE onset are closely associated with right insular infarction (9). The patient developed pulmonary edema within 5 h after cerebral infarction onset. Brain CT showed a large infarction involving the right Ic, and chest CT revealed gravity-dependent pulmonary infiltrates. These findings strongly suggested NPE.

Concurrently, we differentiated it from cardiogenic pulmonary edema. The precipitating factors for cardiogenic pulmonary edema include myocardial infarction, myocardial ischemia, valvular heart disease, arrhythmias, progression of underlying cardiac dysfunction, stress-induced (Takotsubo) cardiomyopathy, fluid overload, severe hypertension, renal failure, and cardiotoxic agents such as alcohol, cocaine, and certain chemotherapeutic agents (10). Cardiogenic pulmonary edema typically progresses through two stages: interstitial pulmonary edema and alveolar pulmonary edema (11). CT findings in interstitial pulmonary edema encompass increased vascular diameter, peribronchovascular interstitial thickening, interlobular septal thickening, and pleural effusion (12). As interstitial edema progresses to alveolar edema, consolidation and ground-glass opacities with central or gravity-dependent distribution become evident (11). The primary mechanism of acute pulmonary edema secondary to acute myocardial infarction is acute left heart failure, characterized by a sudden severe reduction in left ventricular contractility. This leads to a marked decline in cardiac output (CO) and elevated pulmonary venous pressure due to increases in left atrial pressure and left ventricular end-diastolic pressure (13). However, when the patient initially developed pulmonary edema symptoms, the BNP level was <400 pg/mL and the LVEF remained >40%, arguing against acute heart failure and further reducing the likelihood of cardiogenic pulmonary edema (14). Concurrently, the CO was 7.28 L/min, and left ventricular internal dimension at end-systole (LVIDs) and left ventricular internal dimension at end-diastole (LVIDd) were 38 mm (reference: 22–40 mm) and 48 mm (reference: 37–58 mm), respectively, suggesting preserved left ventricular contractile function and normal preload. Pump failure directly results from a reduction in the number of functional myocardial cells following acute myocardial infarction, commonly observed with large infarct size (15, 16). However, typical regional wall motion abnormalities were not detected on either echocardiogram. On the fourth day following the onset of pulmonary edema, although BNP and hs-TnI levels were elevated, they did not fully reflect cardiac functional deterioration. This is because the repeated echocardiogram demonstrated a markedly increased left ventricular end-diastolic volume (LVEDV), which can stimulate BNP synthesis and secretion. However, the left ventricular end-systolic volume (LVESV) showed minimal change, suggesting an improvement in overall systolic function. Combined with the increased LVEF, these findings support improved cardiac function. This rapid improvement in function does not seem to support the presence of extensive myocardial infarction. Undoubtedly, elevated cardiac biomarkers strongly suggested underlying myocardial damage, likely secondary to catecholamine-mediated stress rather than primary cardiac ischemia, as discussed subsequently.

Additionally, the potential association between tenecteplase and pulmonary edema remains unsubstantiated. There have been several reports of acute pulmonary edema occurring after recombinant tissue plasminogen activator (rt-PA) thrombolysis for ischemic stroke (17), It often occurred within one hour post-thrombolysis and was associated with high mortality (18). Studies have demonstrated that rt-PA converts plasminogen to plasmin *in vivo*. This process activates the complement cascade, leading to mast cell degranulation and subsequent release of potent inflammatory mediators. These include histamines, proteases, chemokines, cytokines, and arachidonic acid metabolites, ultimately resulting in angioedema (18). Lugonjic, Sara et al. found that nominal trends for fewer hypotensive and symptomatic intracranial hemorrhage events and more orolingual angioedema events for Tenecteplase compared to rt-PA (19). Although tenecteplase and rt-PA share similar mechanisms, the former exhibits higher fibrin selectivity and reduced systemic effects (20, 21). Furthermore, no direct evidence or published reports currently confirm that tenecteplase can cause acute pulmonary edema. In this case, stroke severity and autonomic dysregulation should be prioritized as primary triggers for consideration, rather than this uncommon and unverified factor. Further research is warranted to investigate whether tenecteplase may indirectly exacerbate pulmonary edema in specific patient populations.

Another question worth consideration is whether Takotsubo syndrome (TTS) exists. TTS is a clinical condition characterized by transient impairment of left ventricular contractility. Patients with TTS typically present with symptoms such as chest pain and dyspnea, ECG changes (ST-segment abnormalities), modest elevation of cardiac biomarkers (CK-MB, troponins, and BNP), and decreased ventricular function, but without evidence of a coronary culprit lesion (22). It is frequently triggered by significant psychological or physical stress. Currently, TTS is thought to share a common pathophysiology with NPE and is sometimes observed concomitantly (23). TTS has historically been regarded as a condition with an exceptionally favorable long-term prognosis, owing to the high likelihood of complete functional recovery (24). A BNP level exceeding 100 pg/mL and an initial troponin level of 0.3 ng/mL have been shown to predict TTS (25). The diagnosis of TTS almost invariably requires coronary angiography to exclude acute coronary syndrome. However, for patients who are hemodynamically stable, without ST-segment elevation on ECG, but with circumferential distribution of dyskinesia on echocardiogram, it is not mandatory (24). The patient exhibited abnormalities in the aforementioned indicators (ECG changes, decreased ventricular function, and elevated CK-MB, hs-TnI, and BNP), all of which rapidly resolved on re-examination within several days. Although lacking coronary angiography, TTS was considered a potential coexisting condition in this patient and a plausible exacerbating factor in the pathogenesis of pulmonary edema.

Notably, the patient developed liver and kidney damage on hospital day 2. This phenomenon is not uncommon among stroke patients. Recent epidemiological data revealed acute kidney injury (AKI) in 9.6% of ischemic stroke patients (26), and abnormal hepatic enzyme levels in approximately 61% at general intensive care unit admission (27). Most peripheral organ damage manifests within the first week after stroke onset, while its direct association with stroke remains inadequately studied to date (27). Some scholars propose that the brain controls various body functions through complex neurohumoral mechanisms. Consequently, any severe cerebral insult, such as acute ischemic stroke, can induce several changes in specific neurosensory or neuromotor pathways, enhance the systemic response to local injury, and cause secondary peripheral organ damage (28). Concurrently, the patient experienced severe pulmonary edema, exhibiting O₂ saturation levels as low as 72.2%, which profoundly compromises peripheral organ function. Hypoxic hepatitis is a common consequence of inadequate oxygen delivery to distal organs, causing a tenfold elevation of AST (29). Three hemodynamic mechanisms are recognized as potential contributors: ischemia due to reduced hepatic blood flow, venous congestion secondary to right heart failure, and hypoxemia from diminished arterial oxygen content (29). Acute pulmonary edema constitutes a common etiology (30). Therefore, hepatic and renal impairment in this patient likely resulted from multiple factors acting in concert, including severe hypoxia secondary to pulmonary edema and neurohumoral regulation following severe brain injury.

## Data Availability

The original contributions presented in the study are included in the article/Supplementary Material, further inquiries can be directed to the corresponding author.
