# Supplementary material for: Case Report: Neurogenic pulmonary edema coupled with myocardial damage following tenecteplase thrombolysis for acute ischemic stroke
Source: Front Cardiovasc Med. 2026 Feb 17;13:1703463. doi: 10.3389/fcvm.2026.1703463 (PMC12953548; doi:10.3389/fcvm.2026.1703463)
Supplement: Supplementary file 1 [file Table1.docx]

Table 1 Selected Echocardiographic Parameters from Two Assessments

| Parameters | EF  (%) | FS  (%) | LVESV  (ml) | LVEDV  (ml) | LVIDd  (mm) | LVIDs  (mm) | LAAP  (mm) | CO  (L/min) | Ventricular wall motion |
| --- | --- | --- | --- | --- | --- | --- | --- | --- | --- |
| Reference (male) | 50-70 | 25-45 | 35-65 | 80-170 | 37-58 | 22-40 | ≤40 | 4-8 | - |
| First  echocardiogram | 41 | 20 | 63 | 107 | 48 | 38 | 50 | 7.28 | Discoordinated |
| Second  echocardiogram | 53 | 27 | 77 | 166 | 58 | 41 | 43 | 6.25 | Discoordinated |

Abbreviations: LVEF: left ventricular ejection fraction; FS: fractional shortening; LVESV: left ventricular end-systolic Volume; LVEDV: left ventricular end-diastolic volume; LVIDd: left ventricular internal dimension at end-diastole; LVIDs: left ventricular internal dimension at end-systole; LAAP: left atrial anteroposterior diameter; CO: cardiac output.
